# Age-Related Changes in Neuromodulatory Control of Bladder Micturition Contractions Originating in the Skin

**DOI:** 10.3389/fnins.2018.00117

**Published:** 2018-02-27

**Authors:** Harumi Hotta, Harue Suzuki, Kaori Iimura, Nobuhiro Watanabe

**Affiliations:** Department of Autonomic Neuroscience, Tokyo Metropolitan Institute of Gerontology, Tokyo, Japan

**Keywords:** electrical stimulation, myelinated nerve fibers, unmyelinated nerve fibers, reflexes, urinary bladder, skin, mechanoreceptors, perineum

## Abstract

The brainstem is essential for producing micturition contractions of the urinary bladder. Afferent input from perineal skin evoked by gentle mechanical stimulation inhibits micturition contractions by decreasing both ascending and descending transmissions between the brainstem and spinal cord. Dysfunction of this inhibitory mechanism may be one cause of the increase in the prevalence of overactive bladder in old age. The aim of this study was to examine effect of aging on function of skin afferent fibers that inhibit bladder micturition contractions in rats. We used anesthetized male rats in three different age groups: young adult (4–5 months old), middle aged (6–9 months old), and aged (27–30 months old). The bladder was expanded to produce isovolumetric rhythmic micturition contractions. Skin afferent fibers were activated for 1 min either by electrical stimulation (0.5 ms, 0.2–10 V, 0.1–10 Hz) of the cutaneous branch of the pudendal nerve (CBPN) or by gentle mechanical skin stimulation with an elastomer roller. When skin afferent nerves were activated electrically, micturition contractions were inhibited in a similar manner in all age groups, with long latency inhibition induced by excitation of Aβ fibers and short latency inhibition by additional Aδ and C fiber excitation (at 1–10 Hz). On the contrary, when skin afferent nerves were activated mechanically by rolling, latency of inhibition following rolling stimulation was prolonged in aged rats. Single unitary afferent nerve activity of low-threshold mechanoreceptors (LTMs) from the cutaneous nerve was recorded. The discharge rate during rolling was not significantly reduced in Aβ units but was much lower in Aδ and C units in aged rats (0.4 and 0.5 Hz, respectively) than in young adult rats (3 and 7 Hz). These results suggest that the neural mechanism that inhibits bladder micturition contractions by skin afferent input is well maintained in old age, but the early inhibition by gentle skin stimulation is decreased because of reduced responses of Aδ- and C-LTMs.

## Introduction

The prevalence of lower urinary tract symptoms such as nocturia, urinary incontinence, and overactive bladder increases with older age (Homma et al., 2006; Irwin et al., 2006; Bosch and Weiss, 2010). Various age-related changes in bladder activity have been reported in humans and other animals. For example, a combination of detrusor overactivity with impaired contraction is seen in bladders in the elderly. In human bladder tissue, cholinergic bladder contractions decrease, while purinergic contractions increase with age (Yoshida et al., 2001). In rats, an increase in bladder volume, a reduced sensitivity of pelvic nerve afferents to bladder volume, and a reduced ability to raise bladder pressure in response to pelvic nerve efferent stimulation are associated with aging (Hotta et al., 1995). Pelvic nerve unmyelinated fibers with smaller diameters decrease in number in aged rats (Nakayama et al., 1998), and pelvic arterial insufficiency has been suggested to play an important role in the development of bladder dysfunctions in humans and other animals (Andersson et al., 2017). However, in addition to such peripheral factors, age-related changes in complex neuronal networks, including the spinal cord and brainstem, regulating bladder function might also be important. The micturition reflex is induced by positive feedback between the bladder and the pontine micturition center (PMC) in the brainstem and is facilitated or inhibited by various sensory inputs. Reduction in the inhibitory mechanism of the micturition reflex may contribute to susceptibility to overactive bladder due to aging. However, the age-related changes in the inhibitory mechanism of the micturition reflex remain to be determined.

In anesthetized adult animals, micturition contractions induced by bladder filling are inhibited by exciting somatic afferent nerves, either by electrical stimulation (Sato et al., 1980; Boggs et al., 2006; Tai et al., 2012; Ferroni et al., 2015) or with natural stimuli (Sato et al., 1975, 1977, 1992; Morrison et al., 1995; Budgell et al., 1998; Hotta et al., 2012). This is because the burst discharges of the pelvic nerve inducing bladder contractions are inhibited by somatosensory input (Sato et al., 1977, 1992, 1997; Hotta et al., 2012). We have recently reported the properties of somatic afferent nerves in the cutaneous branch of the pudendal nerve (CBPN) involved in the inhibition of bladder contraction. The excitation of Aβ fibers at 0.1–10 Hz for 1 min causes late inhibition, emerging several minutes after the end of stimulation, excitation of Aδ fibers at 1–10 Hz produced early inhibition, emerging immediately after stimulus onset, and excitation of C fibers at 1–10 Hz promoted both early and late inhibition (Onda et al., 2016). Similarly, gentle mechanical stimulation of the perineal skin surface with a roller that excites Aβ, Aδ, and C low-threshold mechanoreceptor (LTM) units to discharge at a rate of 2–8 Hz strongly inhibits micturition contraction (Hotta et al., 2012). Perineal rolling inhibits bladder contraction induced by electrical stimulation of the PMC or of the descending tract from the PMC and also inhibits afferent (ascending) transmission from the bladder to the PMC. Therefore, perineal rolling was suggested to shut down the positive feedback between the bladder and the PMC, resulting in inhibition of the micturition reflex (Hotta and Watanabe, 2015). It was shown by randomized clinical trial that perineal rolling reduces the nocturia associated with overactive bladder (Iimura et al., 2016). Such light stimulation to the skin may be applied spontaneously in daily life, and if this inhibitory mechanism is attenuated with age, it may be related to the cause of overactive bladder in the elderly.

The purpose of this study was to examine the effect of aging on the bladder micturition contraction inhibiting function of skin afferent nerve fibers. We employed anesthetized rats of three different age groups and compared the effect of perineal skin stimulation (gentle stimulation with a roller) on micturition contraction. We hypothesized that the inhibitory effect of mechanical skin stimulation is attenuated with age. For comparison, the effects of electrical stimulation of Aβ, Aδ, and C afferent nerve fibers in the skin were also examined. In the present study, age-related changes were observed following only mechanical skin stimulation, so we further examined the possibility of reduced responses of single unitary afferent Aβ-, Aδ-, and C-LTM fibers in the skin of the aged rats.

## Materials and methods

The experiments were performed on 38 male Wistar or Fischer rats. There were no significant differences in their responses, so we combined all data from different strains. The animals were divided into three groups according to their different ages: (1) young adult (4–5 months old, *n* = 15, body weight 260–380 g), (2) middle aged (6–9 months old, *n* = 10, 360–410 g), and (3) aged (27–30 months old, *n* = 13, 330–435 g). The animals were bred at the Tokyo Metropolitan Institute of Gerontology (TMIG) and kept in a specific pathogen-free environment with free access to a commercial pelleted diet and filtered tap water with 2 ppm of chloride. This study was conducted in accordance with the Guidelines for Proper Conduct of Animal Experiments (established by the Science Council of Japan in 2006) and was approved by the animal care and use committee of TMIG. Basic preparation including anesthesia and artificial respiration, recordings of intravesical pressure of micturition contraction, skin stimulation with a roller, electrical stimulation and recordings of unit activity of cutaneous afferents were essentially the same as in our previous studies (Hotta et al., 2012; Onda et al., 2016).

### General surgery

The animals were anesthetized with urethane, after initial inhalation of 3% halothane or 3–4% isoflurane for 2–3 min. During the surgery, 0.3–1.0% halothane or isoflurane was additionally provided as required. An initial dose of urethane was given at 0.9–1.1 g/kg, subcutaneously (s.c.). Additional doses of 0.1–0.2 g/kg were administered intravenously (i.v.), if necessary, to maintain anesthesia at a relatively constant level as judged by the recorded blood pressure. The animals were artificially ventilated via a tracheal cannula to maintain the end-tidal CO_2_ at 3.0–4.0%. Rectal temperature was maintained at 37–38°C by means of an automatically regulated heating pad and lamp (ATB-1100, Nihon Kohden, Tokyo). A jugular vein was catheterized for i.v. administration of supplemental anesthetics and other drugs. A common carotid artery was catheterized to record arterial blood pressure. Rats were euthanized by injecting an overdose of pentobarbital at the end of each experiment.

### Recording of intravesical pressure

A laparotomy was performed, and a catheter was inserted into the bladder via the anterior urethra. The catheter was secured to the urethra by a thread, closing the urethral cavity. To measure intravesical pressure, the urethral catheter was connected to a transducer (TP-200T, Nihon Kohden, Tokyo) via a T-shaped connector. The other end of the T-shaped connector was connected to a syringe pump to manipulate bladder volume. The bladder was filled with saline at a speed of 0.1 ml/min by means of a syringe pump (EP-70, EICOM, Kyoto) connected to the bladder cannula. The saline infusion was stopped when micturition contractions (two or three consecutive contractions) were produced. Then, the micturition contractions continued rhythmically because the urethra had been closed to keep the bladder volume at a suitable range for the micturition reflex (Sato et al., 1992). The frequency of contractions was summarized as a time histogram of contractions counted every 2 min and expressed as contractions per min. Each contraction was counted as one contraction only when its amplitude was above one third of the prestimulus control size.

### Cutaneous stimulation

Gentle mechanical stimulation was applied to the skin of the perineum using a roller with a smooth, soft surface made of elastic polymer (Somaplane, Toyoresin Co., Shizuoka; 17 mm in diameter, 15 mm in length, weighing 4 g), as described previously (Hotta et al., 2012). The hair of the skin area to be stimulated was trimmed with a conventional clipper. The stimulus was applied to a skin area of about 3 cm^2^ for a period of 1 min with a rolling speed of approximately 3 mm/s and a frequency of 10 strokes/min. Rolling was performed manually with a force (roller weight) of 4 g and was paced with an auditory cue.

### Electrical stimulation of cutaneous afferent nerve

After cutting the skin of the lower back on the left side when in the prone position, the CBPN was separated and cut at greater than 20 mm caudal to the sacral plexus. The cavity was kept open by pulling back the edge of the cut skin with threads, and the cavity was filled with warm paraffin oil. The central cut segments of the nerve were placed on bipolar platinum-iridium wire stimulation electrodes, and repetitive rectangular pulses (0.5 ms) were delivered to the nerve. The evoked compound action potential of the CBPN was recorded 17–25 mm proximal from the stimulating site in some cases. During data collection, gallamine triethiodide (20 mg/kg, i.v.) was administered to avoid interference by skeletal muscle activity.

### Recording of unitary afferent nerve activity from perineal skin

Single unitary afferent nerve activity was recorded from CBPN in five young adult rats and four aged rats. The CBPN was separated either in the prone position or the supine position and cut close to the sacral plexus. The peripheral cut segments of the nerve were placed on bipolar platinum-iridium wire recording electrodes. Action potentials of single units were amplified (MEG-2100, Nihon Kohden), audibly monitored through connection to a speaker, visually displayed on an oscilloscope (TS-8500, IWATSU, Tokyo), and digitized (Micro1401, Cambridge Electronic Design, UK) for later processing (Spike 2 software, Cambridge Electronic Design, UK). Receptive fields and mechanical thresholds of each unit were determined using 0.08–4.0 mN von Frey hairs (Touch-test sensory filaments, US Neurologicals). The conduction velocity of each single unit was measured to classify nerve fibers which had conduction velocities >15.6 m/s as Aβ fibers, fibers with conduction velocities between 2 and 15.6 m/s as Aδ fibers, and fibers with conduction velocities <2 m/s as C fibers, as described previously (Hotta et al., 2012).

### Data acquisition and analysis

All analog signals obtained (including intravesical pressure and neuronal potentials) were digitized (Micro1401, Cambridge Electronic Design, UK) for display on a computer monitor and for on-line and off-line analysis using Spike 2 software (Cambridge Electronic Design). Statistical analysis was performed using Prism6 software (GraphPad Software Inc., La Jolla, CA, USA). Values were expressed as mean ± standard error (S.E.). One-way factorial analysis of variance (ANOVA) followed by the Fisher's least significant difference test was used for comparison of values among the three different age groups. The time course of changes in rhythmic micturition contractions induced by somatic stimulation was assessed using repeated measures two-way ANOVA (time and different age groups), followed by Dunnett's multiple comparison test. The properties of single afferent units in aged rats were compared with corresponding data from young adult rats using Student's *t*-test (unpaired). Statistical significance was set at the 5% level.

## Results

### Baseline bladder conditions

Body weight and the basal state of bladder micturition contractions before application of any somatic stimuli were compared between the young adult, middle aged, and aged rat groups (Table 1). Body weight was significantly heavier in middle aged (391 ± 5 g; *p* = 0.0002) and aged (388 ± 11 g, *p* = 0.0004) groups than in the young adult group (332 ± 12 g). However, the bladder volume required to induce micturition contraction was significantly larger in the aged group (1.72 ± 0.30 ml; range 0.6–3.7 ml) than in the young adult (0.96 ± 0.13 ml; range 0.4–1.6 ml, *p* = 0.0083) and middle aged (1.12 ± 0.11 ml; range 0.5–1.6 ml, *p* = 0.0342) groups. Basal pressure, maximum pressure, amplitude, and frequency of micturition contractions did not differ significantly between the three groups. Systolic blood pressure, recorded simultaneously, ranged from 100 to 150 mmHg and did not significantly differ between the three groups.

**Table 1:** Summary of body weight and baseline conditions of the bladders in young adult, middle-aged, and aged rats.

	**Young adult (4–5 months old) *n* = 11**	**Middle aged (6–9 months old) *n* = 10**	**Aged (27–30 months old) *n* = 9**
Body weight (g)	332 ± 12	391 ± 5^**^	388 ± 11^**^
Bladder volume (ml)	0.96 ± 0.13	1.12 ± 0.11	1.72 ± 0.30^**^^,^^#^
Basal pressure (mmH_2_O)	116 ± 7	131 ± 14	137 ± 16
Maximum pressure (mmH_2_O)	526 ± 47	475 ± 56	476 ± 25
Amplitude of contraction (mmH_2_O)	410 ± 45	344 ± 50	339 ± 25
Frequency of contraction (times/min)	1.27 ± 0.18	0.85 ± 0.11	0.83 ± 0.08

Values are mean ± SE;

** *p < 0.01 vs. young adult*,

# *p < 0.05 vs. middle-aged rats, determined by Fisher's least significant difference tests*.

### Inhibition of micturition contractions by electrical stimulation of skin afferent nerves

By recording the strength–response curve of compound action potentials in the CBPN, we confirmed in young adult, middle aged, and aged rats that 0.2 V was suprathreshold for Aβ fibers but subthreshold for Aδ and C fibers, 1.0 V was suprathreshold for Aβ and Aδ fibers but subthreshold for C fibers, and 10 V was suprathreshold for all fibers. Electrical stimulation (pulse duration: 0.5 ms) was applied to the CBPN with these voltages at three different frequencies of 0.1, 1, and 10 Hz for 1 min, and changes in micturition contractions were examined in young adult (*n* = 5), middle aged (*n* = 4), and aged (*n* = 5) rats. The order of nine different stimulus parameters was randomized.

**Figure 1:**
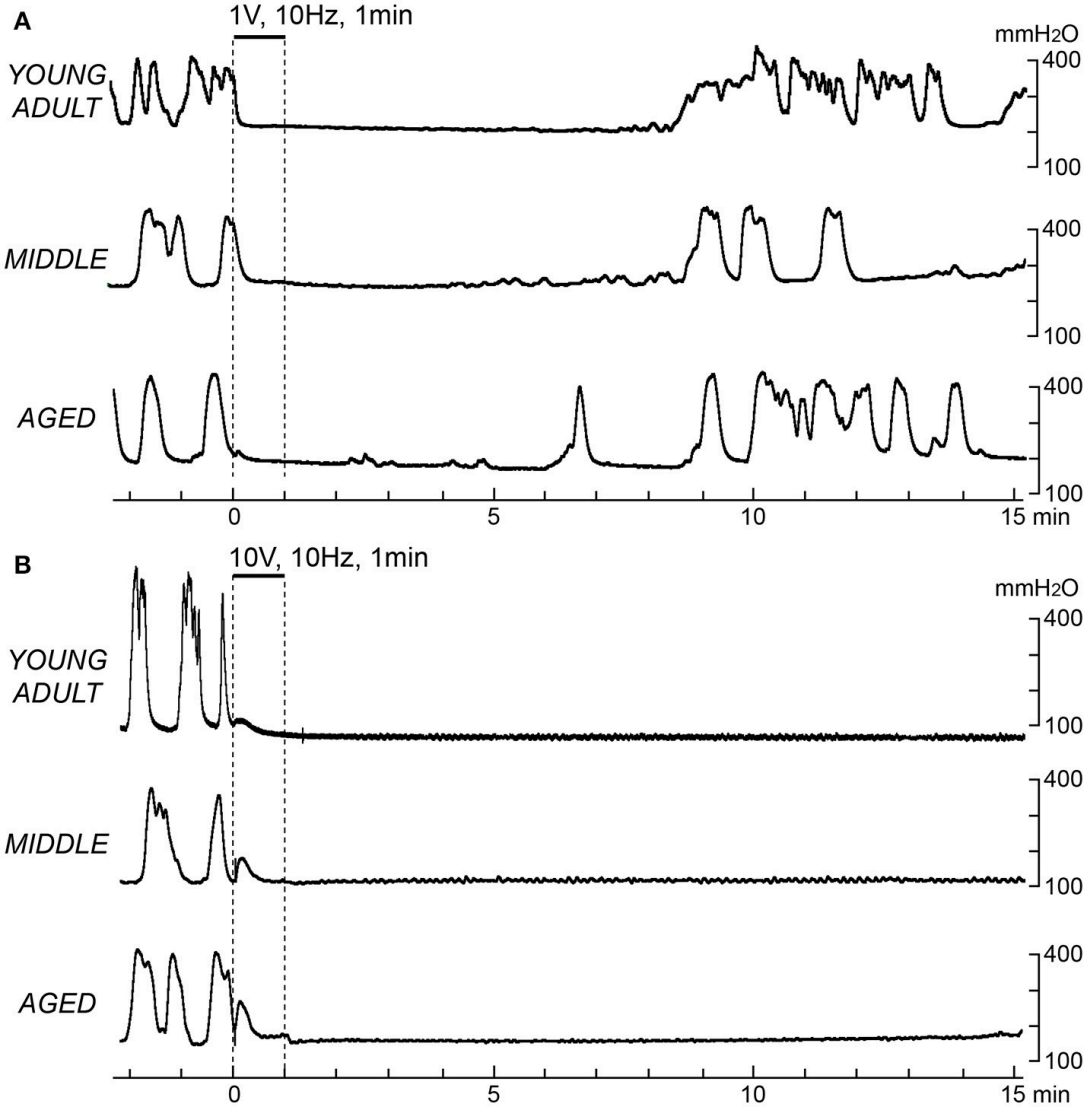
Effects of electrical stimulation of cutaneous branch of the pudendal nerve afferents on rhythmic micturition contractions. Sample recordings in a young adult (upper), a middle-aged (middle), and an aged rat (lower) are shown. Stimulation for 1 min at 10 Hz with 1 V **(A)** and 10 V **(B)** is indicated by the upper horizontal bar and the vertical dashed lines, respectively.

**Figure 2:**
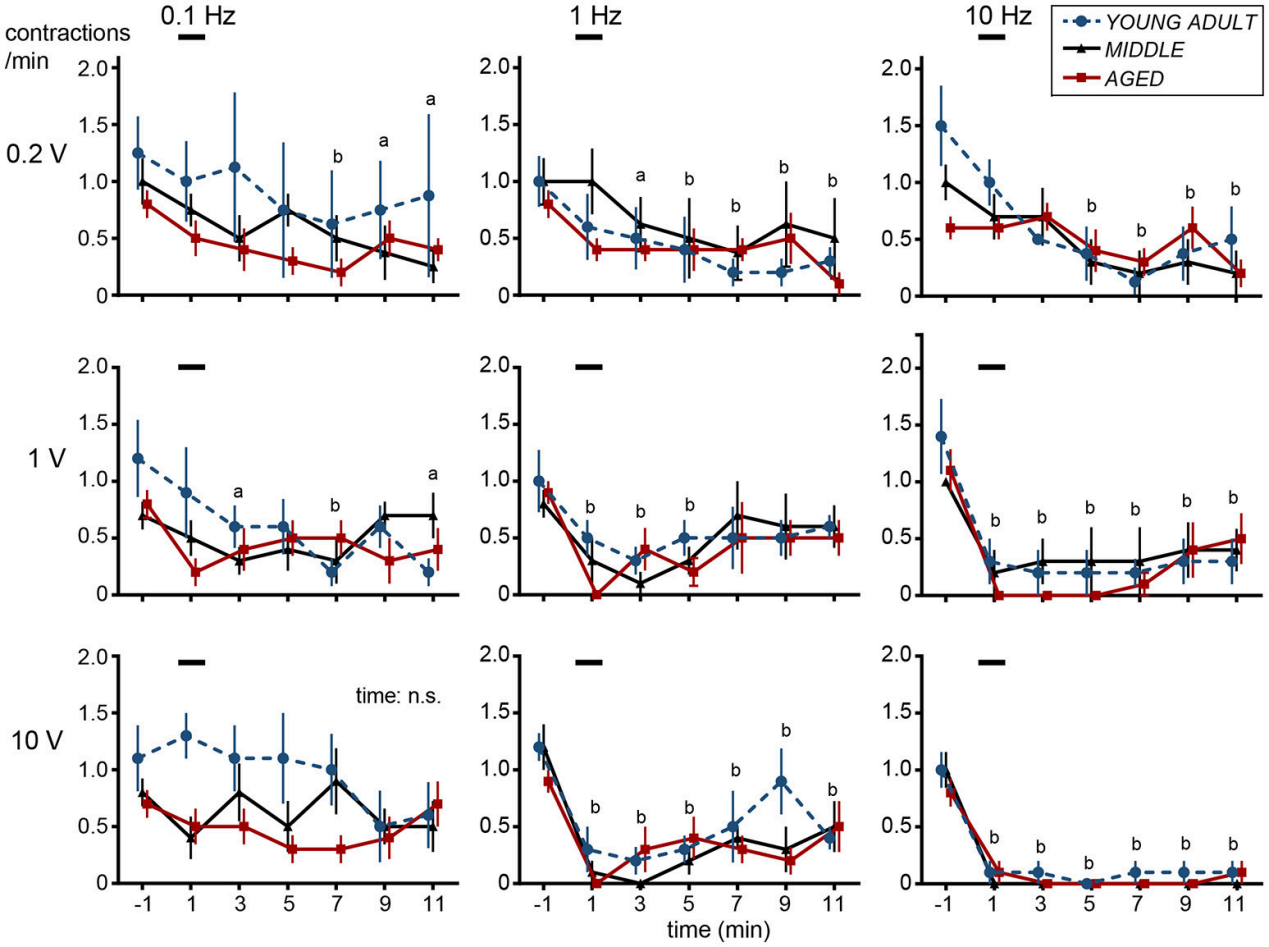
Time histograms of micturition contractions after electrical stimulation of cutaneous branch of the pudendal nerve afferents compiled from five young adult rats (blue circles and dashed lines), four middle-aged rats (triangles and solid lines), and five aged rats (red squares and solid lines). Each rat was subjected to 1–2 trials. Each point represents mean ± SE (4–5 trials from 4–5 rats) for the number of contractions counted every 2 min, expressed as contractions per minute. Onset of stimulation was set as time zero; ^a^*p* < 0.05, ^b^*p* < 0.01, significant differences from prestimulus control values determined by Dunnett's multiple comparison test in pooled data from all three groups.

Figure 1 shows sample recordings in rats of different ages after application of 10-Hz electrical stimulation to CBPN afferents for 1 min with 1 V (Figure 1A) or 10 V (Figure 1B). With 1 V, micturition contractions completely stopped for 6–8 min and then recovered. With 10 V, micturition contractions completely stopped for >15 min. These responses were similar in all age groups. The time course of the effect of electrical stimulation of CBPN at three different voltage intensities (0.2, 1, and 10 V) and three different frequencies (0.1, 1, and 10 Hz) are summarized in Figure 2.

In all nine different stimulus parameters, effects of CBPN stimulation on contraction frequency were similar among different age groups (Figure 2). Two-way ANOVA revealed that the main effect of age was not statistically significant (*p* > 0.19) for all nine stimulus parameters. Interaction was also not significant (*p* > 0.068). However, there were significant time-dependent effects (*p* < 0.019) for eight of the stimulus parameters but not for stimulation at 0.1 Hz with 10-V intensity. Therefore, *post-hoc* tests were performed for the eight parameters on pooled data in all age groups of rats. With 0.2-V intensity at 0.1–10 Hz, the frequency of micturition contractions significantly decreased 7–11 min after the stimulus onset (late inhibition). With 1-V intensity at 1 Hz, the frequency of micturition contractions significantly decreased at 1–5 min (early inhibition) and throughout 1–11 min at 10 Hz (both early and late inhibition). With 10-V intensity at 1–10 Hz, contractions significantly decreased throughout 1–11 min; especially at 10 Hz, contractions were stopped completely in all except one case. Contractions recommenced 14–47 min later in all rat age groups.

### Inhibition of micturition contractions by gentle mechanical skin stimulation

**Figure 3:**
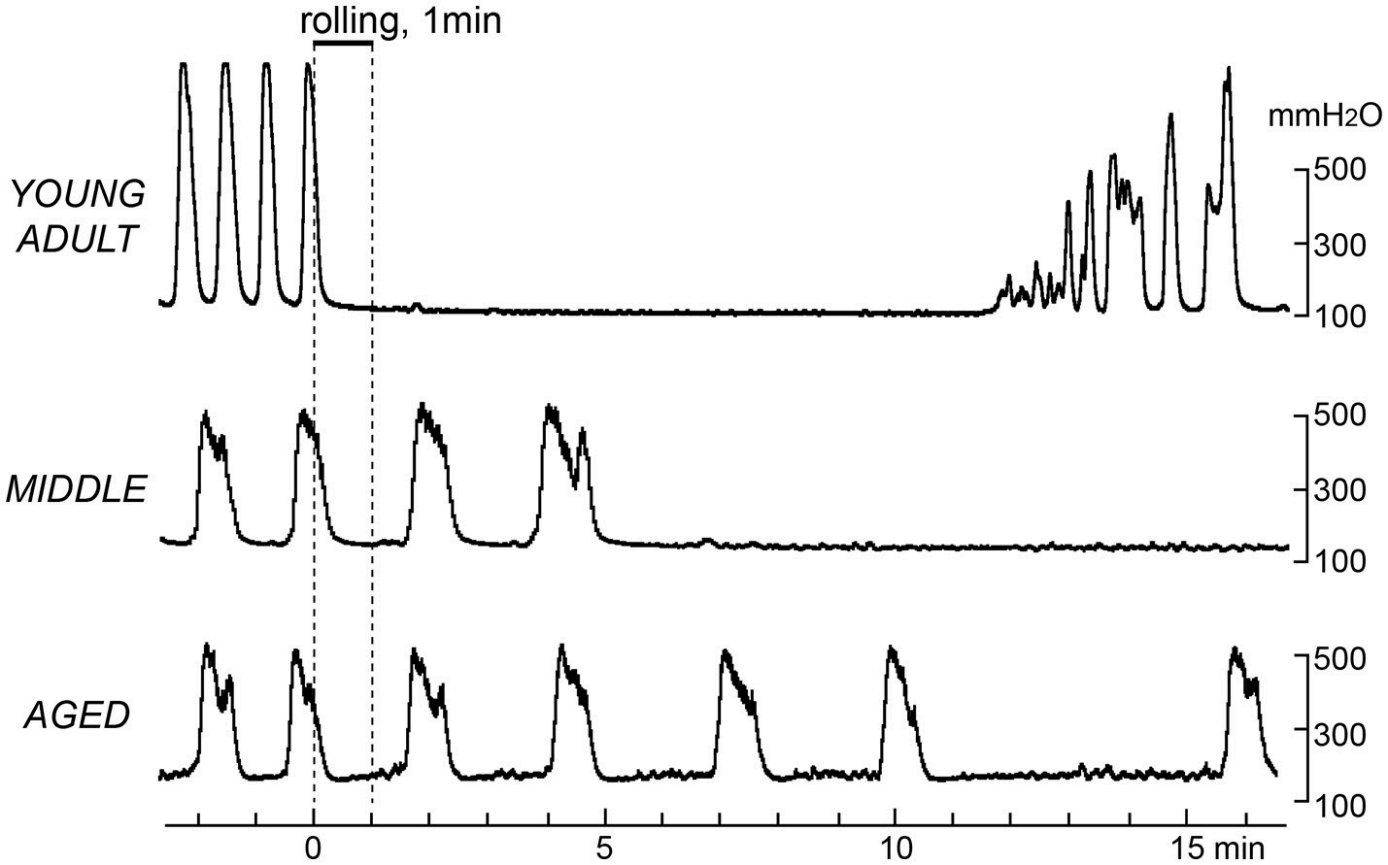
Effects of gentle perineal skin stimulation by a roller on rhythmic micturition contractions. Sample recordings in a young adult **(upper)**, a middle-aged **(middle)**, and an aged **(lower)** rat are shown. Stimulation by perineal rolling for 1 min is indicated by the upper horizontal bar and vertical dashed lines.

Figure 3 shows sample recordings from three rats in the different age groups, in which gentle mechanical stimulation was applied to the perineal skin with a roller for 1 min. Following stimulation, the frequency of micturition contractions decreased in all age groups. However, there was a difference in the time course of inhibition. The onset of inhibition following rolling stimulation of the skin was gradually delayed with age. In a typical case of a young adult rat (upper part of Figure 3), contractions stopped immediately during perineal stimulation and then recovered within 15 min. In a typical case of a middle-aged rat (Figure 3 middle), the contractions continued for several min after stimulation ended and then stopped. In a typical example of an aged rat, the intercontraction interval was gradually increased after stimulation (lower part of Figure 3).

**Figure 4:**
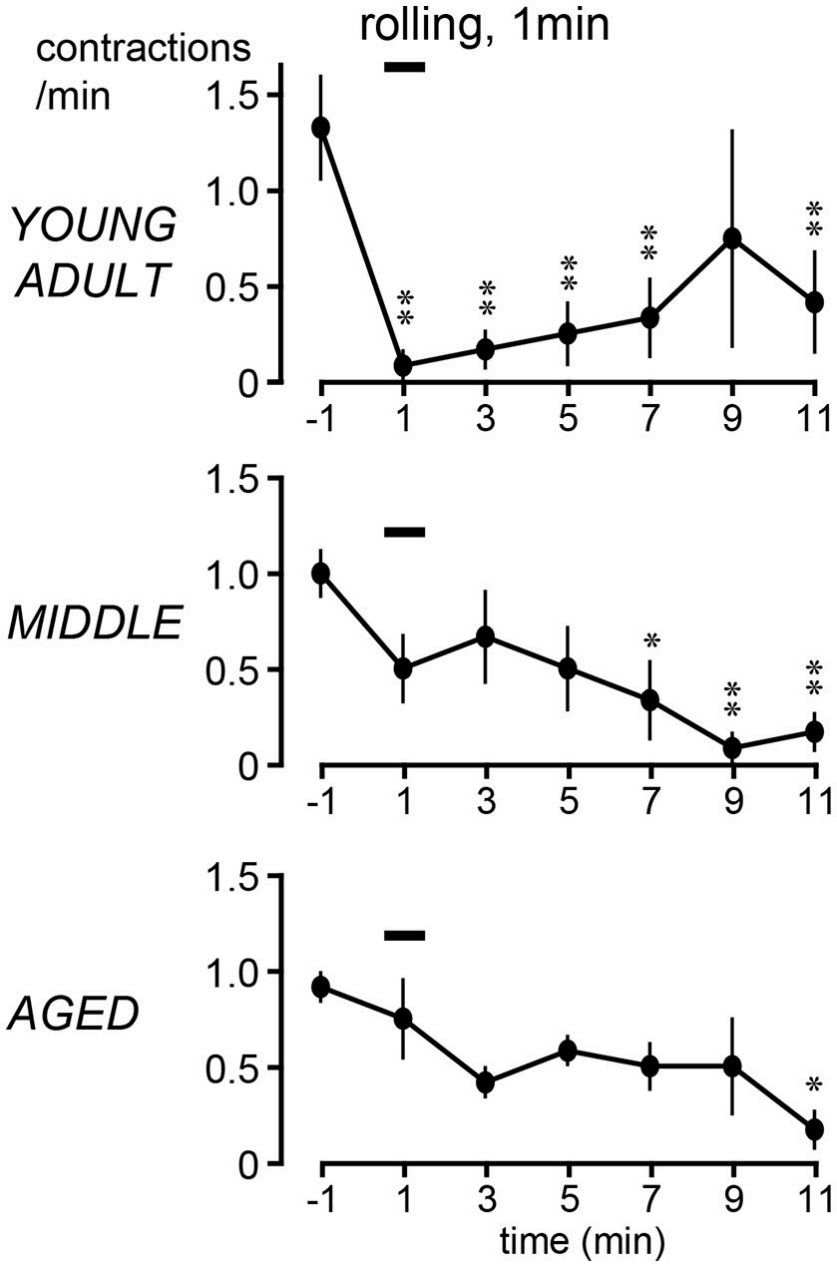
Peristimulus time histograms of micturition contractions after perineal rolling stimulation, compiled from six rats each in young adult (upper), middle-aged (middle), and aged (lower) rats. Details of histogram are same as in Figure 2; ^*^p < 0.05, ^**^p < 0.01, significant differences from prestimulus control values determined by Dunnett's multiple comparison test.

The time course of the effects of rolling stimulation is summarized from six rats for each group in Figure 4, as time histograms of contraction. A significant interaction (*p* = 0.022) was observed when the frequency of micturition contractions was compared between the three groups with two-way ANOVA. The main effect of time was also significant (*p* < 0.0001). However, the main effect of age was not significant (*p* = 0.90). Therefore, *post-hoc* multiple comparison tests of the effect of time were performed for each age group. The *post-hoc* analysis revealed that the time course differed in each group; the decrease in micturition contractions was significant from 1 to 7 and 11 min after stimulation in the young adult group and from 7 to 11 min in the middle-aged group but only at 11 min in the aged group (Figure 4).

**Figure 5:**
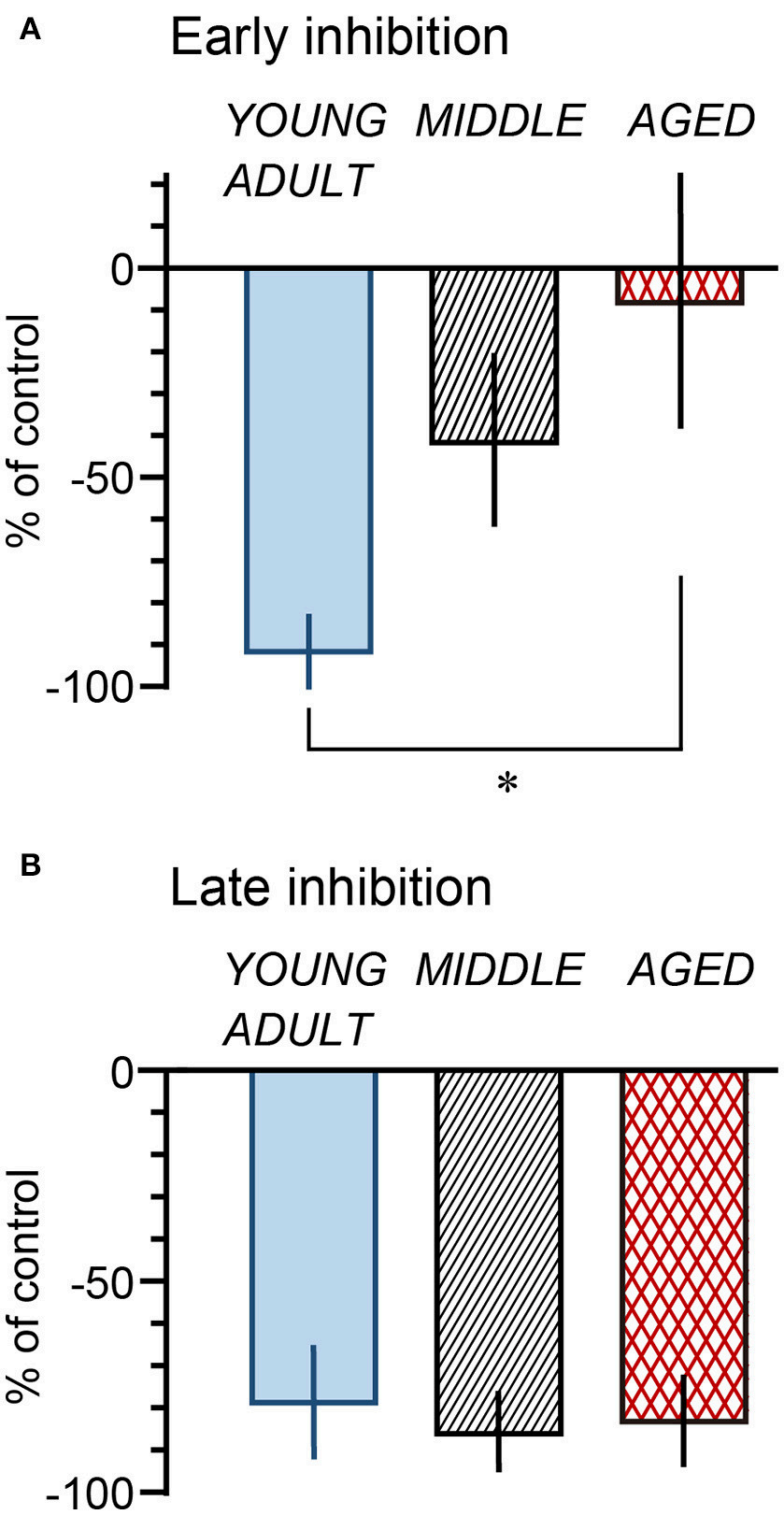
Summary of magnitude of early (**A**: at 1 min) and late (**B**: at 11 min) inhibition by perineal rolling in young adult, middle-aged, and aged rats. Each column and vertical bar indicates the mean ± SE; ^*^p < 0.05, significant differences determined by Fisher's least significant difference test.

The magnitude of the decrease of micturition contractions at 1 min (early inhibition) and 11 min (late inhibition) was expressed as a percentage of the prestimulus control level and compared among the three age groups (Figure 5). The magnitudes of early inhibition were −92% ± 8%, −42% ± 20%, and −8% ± 30% in the young adult, middle-aged, and aged rats, respectively (Figure 5A), gradually reducing with age (*p* = 0.045). The value in the aged rats was significantly lower than that in the young adult rats (*p* = 0.015). The value for the middle-aged rats was not significantly different between the other groups. In contrast, late inhibition (−79 ± 13%, −86 ± 9%, and −83 ± 11% in young adult, middle-aged, and aged rats, respectively) was not significantly different (*p* = 0.90) between the three age groups (Figure 5B). The responses in the aged group, in which late inhibition was predominant, were similar to those induced by selective excitation of Aβ afferents with 0.2-V electrical stimulation.

### Response of single unitary activity of skin afferent LTM fibers

The above results showed that contraction inhibition by skin stimulation was delayed by aging although inhibition by electrical stimulation of CBPN afferents was well maintained. This suggests that the cause of the age-related changes in response to skin stimulation may be due to changes in the function of skin mechanoreceptors. Therefore, firing activity of skin afferent Aβ-, Aδ-, and C-LTM units in response to perineal rolling stimulation was examined in aged rats and compared with the results of young adult rats.

**Figure 6:**
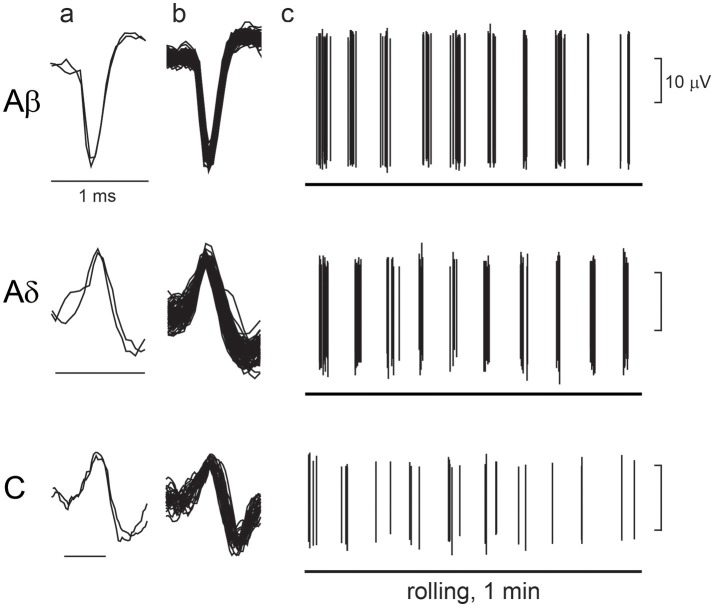
Response of low-threshold mechanoreceptor (LTM) afferent nerve units during rolling. Examples of unitary afferent fiber recordings of Aβ-, Aδ- and C-LTM units in aged rats. **(a)** Action potentials evoked by electrical stimulation of the receptive field. **(b)** Superimposed action potentials recorded during manual rolling stimulation. **(c)** Responses of each unitary afferent fiber to rolling stimulation of the receptive field skin by a roller. A horizontal line indicates the period of rolling stimulation.

Figure 6 shows an example recording of single unitary afferent nerve activities of Aβ-, Aδ-, and C-LTM units in aged rats responsive to the perineal rolling stimulus. The conduction velocity of each unit was 16.1, 7.3, and 0.94 m/s, respectively. In each of these units, periodic activities synchronized with the movement of the rollers on the receptive field were observed. The average discharge frequency of Aβ, Aδ, and C fiber action potentials during stimulation for 60 s was 2.4, 1.5, and 0.5 Hz, respectively.

A total of 26 and 23 units were recorded in young adult and aged rats, including 12 and 9 units classified as Aβ fibers, 9 and 9 units classified as Aδ fibers, and 5 and 5 units classified as C fibers, respectively. In young adult and aged rats, the conduction velocities of Aβ fibers were 27.4 ± 1.6 m/s (with a range of 19.0–37.9 m/s) and 23.6 ± 1.6 m/s (16.1–30.0 m/s), Aδ fibers were 9.6 ± 1.1 m/s (5.5–15.3 m/s) and 8.6 ± 0.8 m/s (4.2–12.4 m/s), and C fibers were 0.90 ± 0.12 m/s (0.6–1.3 m/s) and 0.87 ± 0.17 m/s (0.43–1.35 m/s), respectively. All values in aged rats were equivalent to those in young adult rats.

The von Frey thresholds measured in 21 units from aged rats were within the range of 0.08–4 mN, which was the same as those in 24 units from young adult rats. However, in young adult rats, variations of the threshold in Aβ-, Aδ-, and C-LTM units were equal, whereas in the aged group, all C-LTM units responded to the lowest level of 0.08 mN stimulation.

**Figure 7:**
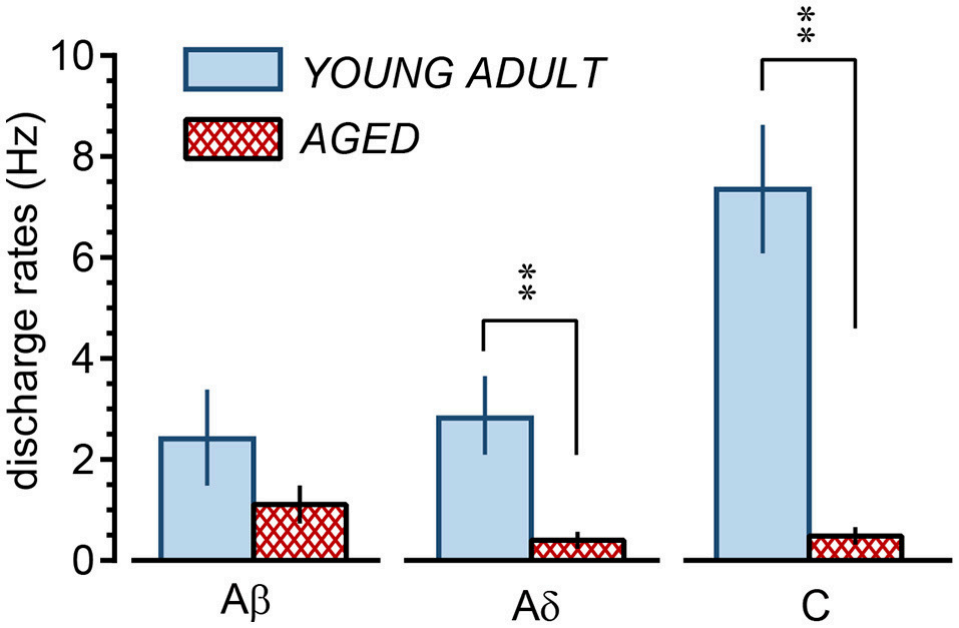
Summary of discharge rates of low-threshold mechanoreceptor afferent nerve fibers during perineal rolling (1 min) in young adult and aged rats. Each column and vertical bar indicates the mean ± SE; ^**^p < 0.01, significant differences determined by Student's *t*-test.

In the young adult and aged rats, the mean discharge rates during cutaneous stimulation of the Aβ fibers were in the range of 0.03–11.2 (2.4 ± 0.9) Hz and 0.08–2.8 (1.1 ± 0.4) Hz, respectively (Figure 7), a slight but not significant (*p* = 0.24) reduction for the aged rats. However, the mean discharge rates during cutaneous stimulation of the Aδ fibers were 0.1–7.3 (2.9 ± 0.8) Hz and 0.02–1.5 (0.4 ± 0.2) Hz in the young adult and aged rats, respectively, showing significant reduction (*p* = 0.0078) in aged rats. Further, the mean discharge rates during cutaneous stimulation of the C fibers were 3.3–11.1 (7.3 ± 1.3) Hz in young adult rats, the highest among three fiber groups, but were much reduced to 0.1–1.1 (0.5 ± 0.2) Hz in aged rats (*p* = 0.0007; Figure 7).

## Discussion

In the present study, the inhibition of micturition contractions in response to gentle mechanical cutaneous stimulation delayed with age. The weaker bladder inhibition may be due to changes in the peripheral or central nervous system, bladder, or skin mechanoreceptors. However, inhibition in response to electrical stimulation of the skin afferent nerve was well maintained in aged rats and was similar to that in adult rats. Therefore, age-related functional changes in the peripheral or central nervous system or bladder do not appear to be involved in the changes in contraction inhibition. Moreover, the discharge rate of skin Aδ- and C-LTM afferent fibers during gentle mechanical stimulation was markedly reduced in aged rats compared to that in adult rats. These results suggest that age-related functional changes in skin mechanoreceptors, specifically in Aδ and C fibers that trigger early inhibition, are responsible for the delay of contraction inhibition.

### Basal condition of the aged bladder

The basal pressure, maximum pressure, amplitude, and frequency of micturition contractions were not significantly different between the young adult, middle-aged, and aged groups. However, the bladder volume inducing micturition contractions was about 70% larger in aged (27–30 months old) than in young adult (4–5 months old) and middle-aged (6–9 months old) rats. The results showing the increase in bladder capacity in aged rats are consistent with previous results comparing cystometrograms of young adult rats of 2–3 months old with aged rats of 26–29 months old. In that study, the volume at an intravesical pressure of 200 mmH_2_O was 1.75 ± 0.26 ml in aged rats vs. 0.33 ± 0.05 ml in young-adult rats, and the cystometrograms were significantly shifted to the right in the aged rats (Hotta et al., 1995). Zhao et al. also reported that conscious, freely moving, old (28–30 months old) rats had increased bladder capacity, post-void residual volume, baseline, and intermicturition pressure but decreased micturition pressure compared with young (4–6 months old) rats. These changes were associated with decreased muscle mass and increased collagen deposition in the old bladder (Zhao et al., 2010). Recently, age-related increases in the collagen-smooth muscle ratio were also reported in old (85-week-old) mice using *ex vivo* two-photon laser scanning microscopy (Schueth et al., 2016).

### Inhibition by electrical stimulation of perineal skin afferents

The stimulation intensity and frequency dependence of inhibition of micturition contractions due to electrical stimulation in aged rats was essentially maintained as in younger adult rats. This result suggests that both the peripheral and central neural mechanisms involved in the somato-vesical inhibitory reflex are maintained in aged rats. Late inhibition was induced by activation of only Aβ fibers over a wide range of 0.1–10 Hz, but excitation of Aδ or C fibers at 1–10 Hz was required to induce early inhibition. These features are in accord with a previous study in adult rats (Onda et al., 2016). Complete inhibition by 10 V, 10-Hz stimulation was shown not to be affected by blocking capsaicin-sensitive C fibers in adult rats (Onda et al., 2016). The present results indicate that the mechanisms for late inhibition by excitation of Aβ fibers, early inhibition by additional excitation of Aδ fibers, and early and late inhibition by further excitation of C fibers, are all well-maintained during aging. Treatment of overactive bladder by electrical stimulation is used clinically, and many of the patients are elderly (Guo et al., 2014). The influence of age on the inhibition of micturition contractions induced by electrical stimulation has not previously been reported. Our results suggest that electrical stimulation therapy may be effective for the elderly, as for younger adults.

### Inhibition by gentle stimulation of perineal skin

In contrast to the well-maintained inhibition of micturition contractions by electrical stimulation, inhibition by gentle mechanical skin stimulation changed with age. Although the inhibitory effect itself was maintained, the latency of inhibition was prolonged with age. The inhibition of micturition contractions following cutaneous rolling in the aged group was similar to that following electrical stimulation of only Aβ fibers. By recording the LTM unitary activity, the response of Aβ-LTM units during rolling was maintained in the aged group, but Aδ- and C-LTM unitary activity during rolling in aged rats was much lower than in adult rats. Mean discharge rates of Aδ- and C-LTM units in young adult rats were 3–7 Hz, whereas those in aged rats were 0.4–0.5 Hz. Therefore, considering that electrical stimulation of Aδ and C fibers at frequencies of 1–10 Hz was necessary for inducing early inhibition, lack of the early inhibition by skin stimulation in aged rats appears to be due to reduced responses of Aδ- and C-LTMs, and the late inhibition observed in aged rats appears to be caused by activity of Aβ-LTMs.

It has been shown in adult rats that rolling of the skin inhibits PMC neuronal activity induced by bladder distension and also inhibits bladder contraction by PMC stimulation (Hotta and Watanabe, 2015). The inhibitory effect on PMC activity may be delayed in aged rats, resulting in a delay of inhibition of micturition contractions. In humans, rolling stimulation, self-applied before bedtime for 1 min, has been shown to alleviate nocturia caused by overactive bladder in the elderly (Iimura et al., 2016). As a clinical effect of the rolling on nocturia, late inhibition rather than early inhibition would be important. However, the lack of rapid inhibition by gentle skin stimulation may be related to urge incontinence, which increases with age.

### Age-related changes in skin mechanoreceptors

Tactile sensations involving Aβ fibers, such as sensing vibration, and the density of Pacinian corpuscles and Meissner bodies connecting to Aβ fibers decreases with age (reviews of Wickremaratchi and Llewelyn, 2006; Decorps et al., 2014). Although only one report has examined the function of single afferent Aβ units, there were no differences in the receptive field sizes and von Frey thresholds in the planter nerve of 6- and 24- to 27-month-old rats (Reinke and Dinse, 1996). Their result on hairless skin is consistent with our result on hairy skin. However, there have been no reports investigating age-related changes in single unitary activities of small diameter LTM fibers from the skin. Our study showed for the first time that the responses of Aδ- and C-LTM units are selectively decreased with age.

Aδ-LTMs and C-LTMs are abundantly present in the hairy skin of humans and animals (Adriaensen et al., 1983; Djouhri, 2016) and suggested to project to the limbic cortices (Olausson et al., 2002; Watanabe et al., 2013) but do not contribute to tactile sensation. This contrasts with Aβ fibers, which project to the neocortical primary somatosensory cortex and contribute to tactile sensation. Inhibition of the somato-cardiac sympathetic C-reflex by gentle touch (Hotta et al., 2010; Watanabe et al., 2012), mainly caused by activity of Aδ- and C-LTMs (Watanabe et al., 2015), was attenuated in aged rats (Watanabe et al., 2011). On the contrary, inhibition of adrenal sympathetic nerve activity by brushing, mainly caused by activity of Aβ- and Aδ-LTMs (Isa et al., 1985), was well maintained in the aged rats (Kurosawa et al., 1987). These different cutaneous effects of aging may be explained by our results showing the attenuated responses of Aδ- and C-LTMs and the well-maintained responses of Aβ-LTMs.

The frequency of discharges to mechanical stimuli and the size of the receptive fields of LTMs, including unmyelinated tactile afferents, generally depend on the strength of the indentation (Wessberg et al., 2003), and if the mechanical threshold is higher, the frequency of discharge in response to the same 4-g roller stimulus would lower. However, it is of note that for Aδ- and C-LTM units in aged rats, the von Frey threshold was not higher, but the response frequency during rolling was much lower than that in younger adult rats. This may be due to receptor-specific changes that evoke afferent volleys in Aδ- and C-LTMs, such as a decrease in the number and/or changes in the properties of fine Zigzag hairs in which Aδ- and C-LTMs are selectively distributed (Abraira and Ginty, 2013). It will be important to clarify the mechanisms of age-related changes in responses of Aδ- and C-LTMs in future studies.

## Conclusion

In summary, we reported three important age-related results in response to gentle mechanical cutaneous and electrical stimulation: (1) inhibition of micturition contractions induced by gentle mechanical stimulation had a delayed onset, (2) discharge rate of Aδ and C-LTM skin afferent fibers was markedly reduced, and (3) inhibition of micturition contractions induced by electrical stimulation was well maintained during aging. Electrical stimulation can maintain the firing rate of the cutaneous afferent, which is different from mechanical rolling stimulation (Table 2). Therefore, we can conclude that the reduced firing rate in skin mechanoreceptors during mechanical stimulation is the cause of weak inhibition observed in aged rats study.

**Table 2:** Summary of the present results.

**Skin afferent fiber type**	**Electrical stimulation**		**Gentle mechanical stimulation**	
	**Afferent activity (Hz)**	**Effect on bladder**	**Afferent activity (Hz)**	**Effect on bladder**
**(A) YOUNG ADULT**				
Aβ fiber	0.1–10	Late inhibition	2	Early and Late inhibition
Aδ fiber	1–10	Early inhibition	3	
C fiber	1–10	Early and Late inhibition	7	
**(B) AGED**				
Aβ fiber	0.1–10	Late inhibition	1	Late inhibition
Aδ fiber	1–10	Early inhibition	0.4	
C fiber	1–10	Early and Late inhibition	0.5	

The autonomic nerve itself is relatively resistant to aging (Hotta and Uchida, 2010), but the aging processes affecting tactile function begin from an early age (Verrillo, 1980; Giuseppe et al., 1994; Stevens and Patterson, 1995). This study showed for the first time the possibility that age-related changes in skin function may affect brainstem functions regulating visceral activities.

## Author contributions

HH contributed to study design, data acquisition, data analysis, data interpretation and manuscript writing. HS contributed to data analysis, data interpretation and manuscript writing. KI contributed to data acquisition, data analysis and manuscript revising. NW contributed to study design, data interpretation and manuscript writing. All authors approved the final version of the manuscript and agreed to be accountable for all aspects of the work in ensuring that questions related to the accuracy or integrity of any part of the work are appropriately investigated and resolved.

### Conflict of interest statement

The authors declare that the research was conducted in the absence of any commercial or financial relationships that could be construed as a potential conflict of interest.
